# Proteomic discovery of substrates of the cardiovascular protease ADAMTS7

**DOI:** 10.1074/jbc.RA119.007492

**Published:** 2019-03-29

**Authors:** Alain Colige, Christine Monseur, James T. B. Crawley, Salvatore Santamaria, Rens de Groot

**Affiliations:** From the ‡ Laboratory of Connective Tissue Biology, GIGA, University of Liège, Sart-Tilman, 4000 Liège, Belgium and; § Centre for Haematology, Imperial College London, W12 0NN London, United Kingdom

**Keywords:** ADAMTS, substrate specificity, proteolysis, tissue inhibitor of metalloproteinase (TIMP), proteolytic enzyme, ADAMTS7, autolysis, cleavage sites, LTBP4, TIMP-4

## Abstract

The protease ADAMTS7 functions in the extracellular matrix (ECM) of the cardiovascular system. However, its physiological substrate specificity and mechanism of regulation remain to be explored. To address this, we conducted an unbiased substrate analysis using terminal amine isotopic labeling of substrates (TAILS). The analysis identified candidate substrates of ADAMTS7 in the human fibroblast secretome, including proteins with a wide range of functions, such as collagenous and noncollagenous extracellular matrix proteins, growth factors, proteases, and cell-surface receptors. It also suggested that autolysis occurs at Glu-729–Val-730 and Glu-732–Ala-733 in the ADAMTS7 Spacer domain, which was corroborated by N-terminal sequencing and Western blotting. Importantly, TAILS also identified proteolysis of the latent TGF-β–binding proteins 3 and 4 (LTBP3/4) at a Glu-Val and Glu-Ala site, respectively. Using purified enzyme and substrate, we confirmed ADAMTS7-catalyzed proteolysis of recombinant LTBP4. Moreover, we identified multiple additional scissile bonds in an N-terminal linker region of LTBP4 that connects fibulin-5/tropoelastin and fibrillin-1–binding regions, which have an important role in elastogenesis. ADAMTS7-mediated cleavage of LTBP4 was efficiently inhibited by the metalloprotease inhibitor TIMP-4, but not by TIMP-1 and less efficiently by TIMP-2 and TIMP-3. As TIMP-4 expression is prevalent in cardiovascular tissues, we propose that TIMP-4 represents the primary endogenous ADAMTS7 inhibitor. In summary, our findings reveal LTBP4 as an ADAMTS7 substrate, whose cleavage may potentially impact elastogenesis in the cardiovascular system. We also identify TIMP-4 as a likely physiological ADAMTS7 inhibitor.

## Introduction

**Figure 1.**
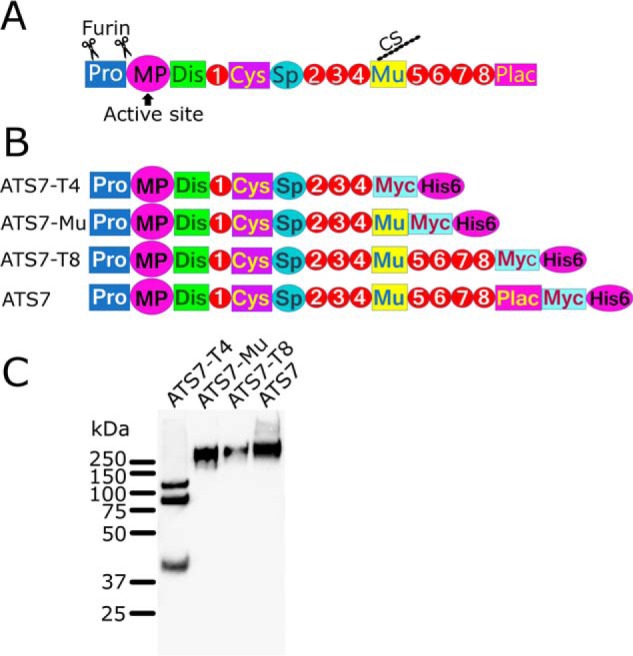
**ADAMTS7 constructs.** *A*, domain organization of ADAMTS7. The ADAMTS7 zymogen contains a Prodomain that maintains latency until, upon secretion, furin cleaves at two sites in the Prodomain to generate the mature active form. The MP domain contains the active site. The ancillary domains consist of eight thrombospondin type 1 domains (numbered), a cysteine-rich domain (Cys), Spacer (*Sp*), mucin-like (Mu) and a protease and lacunin (*PLAC*) domain. The mucin-like domain has a chondroitin sulfate (*CS*) chain attached. *B*, recombinant ADAMTS7 and truncated variants were generated with a C-terminal Myc and His_6_ tag. *C*, Western blotting of recombinant ADAMTS7 and truncated variants, detected with an anti-myc tag Ab. The expression of full-length ADAMTS7 was very poor and conditioned medium was concentrated ∼100× for visualization on Western blotting.

ADAMTS7 is an extracellular metalloprotease and 1 of 19 human ADAMTS family members (1). It is a large protein (>250 kDa), consisting of 15 domains (Fig. 1*A*). A Prodomain, located N-terminal to the metalloprotease (MP)^2^ domain, is predicted to maintain latency until it is removed by proprotein convertases. The MP domain is typical for that of metzincin metalloproteases, which contain an active site of three histidines and a catalytic glutamic acid (2). The domains C-terminal of the MP domain most likely provide exosites that enhance substrate binding and specificity, based on studies of other ADAMTS family members (3–5).

The physiological function of ADAMTS7 is not known, but recent evidence shows that it functions in the extracellular matrix (ECM) of cardiovascular tissues (6–10). Analysis of normal expression of ADAMTS7 in healthy adult tissues shows it is predominantly expressed in the heart and in the tunica media of the lung vasculature, but also at lower levels in other tissues, including tendon (1, 7, 11). In the arterial wall, ADAMTS7 is up-regulated in response to injury/inflammation (7, 8). In this setting, it influences vascular smooth muscle cell migration, possibly by affecting the composition/integrity of the ECM and/or by influencing the availability of growth factors. Genome Wide Association Studies have established *ADAMTS7* as a susceptibility locus for coronary artery disease (CAD) (6, 12) and atherosclerosis is reduced in *Adamts7*^−/−^ mice (7). ADAMTS7 has been detected around vascular smooth muscle cells (and macrophages) in human atherosclerotic plaques, and elevated ADAMTS7 staining in the vessel wall has been associated with a high-risk plaque phenotype (7, 9, 13).

Physiologically, protease activity is often regulated by endogenous inhibitors. Several metalloprotease families are regulated by the tissue inhibitor of metalloprotease (TIMP) family of inhibitors, which in humans consist of four members (TIMP1–4) (14). Whereas all four TIMPs inhibit many members of the matrix metalloprotease family, ADAMTS1, 2, 4, and 5 are only inhibited by TIMP-3 (14). For ADAMTS7, like most other ADAMTS family members, inhibition by TIMPs has not been investigated.

The substrate specificity of ADAMTS7 is poorly characterized. Only two proteins, cartilage oligomeric matrix protein (COMP) and thrombospondin 1, have previously been reported as substrates, without identification of cleavage sites (10, 15). Moreover, a comprehensive analysis of the substrate repertoire and cleavage sites of ADAMTS7 has not been conducted. Knowledge of the cleavage sites is important for the understanding of the functional consequences of proteolysis. It can also guide the design of (therapeutic) inhibitors that derive selectivity and potency from mimicking substrate residues flanking the cleavage site. It is essential to identify the physiological targets of ADAMTS7 to enable a causal link to be established between ADAMTS7 function and CAD/atherosclerosis. To this end, we used terminal amine isotopic labeling of substrates (TAILS), which is a method that employs the labeling of neo–N termini generated by proteolysis to identify and quantify cleavage products by MS-based proteomics (LC-MS/MS) (16).

## Results

### TAILS analysis

To study the substrate specificity of ADAMTS7, we employed TAILS. For this, recombinant ADAMTS7 and truncated variants thereof were generated and expressed (Fig. 1). The truncated variant ADAMTS7-Mu, which consists of the first 10 N-terminal domains, was expressed at the highest levels and was therefore used in this study. As a control for proteolysis, we generated an inactive variant, ADAMTS7-Mu(E389Q) in which the glutamic acid in the active site of the MP domain (Glu-389) is mutated to glutamine, a mutation commonly made to render metzincin proteases inactive (2). As a source of endogenously expressed extracellular matrix substrates we used human fibroblasts. These were co-cultured with HEK cells stably transfected with either ADAMTS7-Mu or the inactive ADAMTS7-Mu(E389Q) for 48 h and the conditioned medium was used for iTRAQ labeling of new N termini that are generated by proteolysis. Analysis of the LC-MS/MS results identified 45 extracellular and transmembrane proteins that exhibited evidence of increased proteolysis in the presence of ADAMTS7-Mu *versus* ADAMTS7-Mu(E389Q) (Table S1). This list of candidate substrates includes proteins with a wide range of functions, such as collagenous and noncollagenous ECM proteins, growth factors, proteases, and cell surface receptors. Interestingly, ADAMTS7 itself was among the candidate substrates (Table S2), suggesting autolytic reactions. Although enrichment of neo–N termini in the presence of active ADAMTS7 suggests that the new N terminus results from proteolysis by ADAMTS7, targets require verification, as indirect effects such as the up-regulation or activation of other proteases provide alternative explanations.

### Autolysis at Glu-Ala/Val bonds

**Figure 2.**
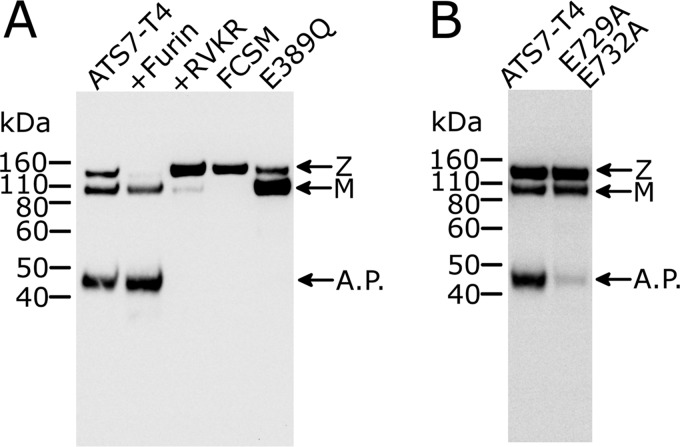
**Western blotting of ADAMTS7-T4 confirms autolysis as revealed by TAILS.** *A*, autolysis can be prevented either by inactivating ADAMTS7 or by abolishing activation by furin. Western blotting (reducing) of conditioned medium containing ADAMTS7-T4, detecting with anti myc-tag Ab, shows zymogen (*Z*), a mature (*M*) form, and a 45-kDA autolytic product (*A.P.*). Conditioned medium was loaded after 2-h incubation with either buffer (*lane 1*) or 50 nm recombinant furin (*lane 2*). Decanoyl-RVKR-CMK is an inhibitor of furin which was added to the medium (10 μm) post transfection to prevent conversion of the latent zymogen to its active (mature) form (*lane 3*). Activation was also abolished in the FCSM (R68A/R70A/R232A/R236A) (*lane 4*). The mutation E389Q in the active site of the metalloprotease domain abolishes proteolytic activity and, consequently, the appearance of the 45-kDa autolytic product (*lane 5*). *B*, the P1 residues of the autolytic cleavage sites identified by TAILS and N-terminal sequencing were mutated to alanine in ADAMTS7-T4.

To verify that ADAMTS7 cleaves itself, as suggested by TAILS, we reassessed the Western blotting of one of the truncated ADAMTS7 variants, ADAMTS7-T4, which also showed potential evidence of autolysis (Fig. 1*C*). This variant lacks the highly glycosylated mucin-like domain, which greatly reduces the molecular weight (MW) of any C-terminal fragment arising from autolysis that can be detected with an anti-myc tag antibody (Ab). On Western blotting of ADAMTS7-T4, detecting with an anti-myc Ab, a band corresponding to the zymogen (>110 kDa), which has the Prodomain still attached, was resolved from the mature, active form (<110 kDa), which has the Prodomain removed (Fig. 2*A*). An unexpected, low-MW band of 45 kDa, we hypothesized, could be a product of autolysis. This band would correspond to a C-terminal cleavage product generated by autolysis in the Spacer domain. To confirm the identity of these bands, ADAMTS7-T4 was incubated with furin, a subtilisin-like proprotein convertase which is responsible for Prodomain removal in the ADAMTS family (1, 17). The >110-kDa band, corresponding to the ADAMTS7 zymogen, disappeared after incubation with furin (Fig. 2*A*). In addition, the mature form (<110 kDa) and the suspected autolytic cleavage product (45 kDa), were both absent when the furin inhibitor Decanoyl-RVKR-CMK was added during expression. Moreover, a mutant in which the furin cleavage sites were mutated, FCSM (R68A/R70A/R232A/R236A), also showed the >110-kDa band (zymogen) only, which confirms that the Prodomain maintains latency and thus prevents generation of the 45-kDa autolytic cleavage product. The inactive ADAMTS7-T4(E389Q) variant also lacked the 45-kDa band, further confirming its generation depends on ADAMTS7 activity.

To identify the scissile bond that is targeted in this autolytic reaction, we isolated the 45-kDa cleavage product for N-terminal sequencing. The N-terminal sequence was identified as “AANFL,” which matches an abundant autolytic product identified by TAILS (AANFLALR) (Table S2). This new N terminus is generated by autolysis at the Glu-732–Ala-733 bond in the Spacer domain. Interestingly, the adjacent and similar Glu-729–Val-730 bond is also a major target, as demonstrated by abundant VAEAANFLALR peptide identified by TAILS.

As both the Glu-729–Val-730 and the Glu-732–Ala-733 bonds contain a glutamic acid residue in the P1 position, we mutated these to alanine to investigate the preference of ADAMTS7 for a glutamic acid residue in P1. The mutant ADAMTS7-T4(E729A/E732A) showed markedly reduced autolysis (Fig. 2*B*), showing that glutamic acid residues are better accommodated in the S1 pocket of ADAMTS7, compared with alanine. However, a faint 45-kDa band was still visible, showing that Ala in P1 permits proteolysis, but at a reduced rate.

**Figure 3.**
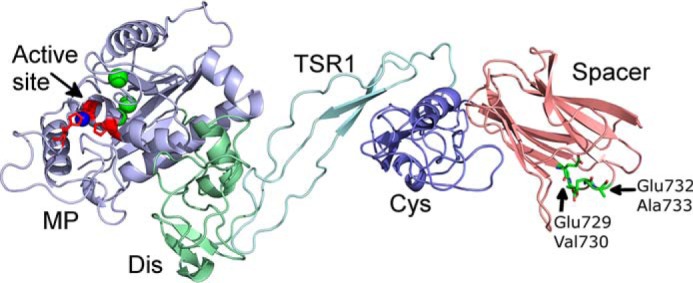
**Structural model of ADAMTS7 MP-Spacer domains.** The model of the ADAMTS7 Spacer domain structure revealed that the autolytic cleavage sites identified by N-terminal sequencing and TAILS (Glu-729–Val-730 and Glu-732–Ala-733) are present in the surface exposed β3-β4 loop of the Spacer domain. The active-site zinc in the MP domain is shown in *blue*, structural calcium ions are shown in *green*. The structure of the N-terminal domains of ADAMTS7 (MP-Spacer) was modeled using the Dis-Spacer structure of ADAMTS13 (3GHM) and ADAMTS1, 4, and 5 MP domain structures (Protein Data bank entries 2RJQ, 2RJP, 4WK7, 2V4B).

To visualize the location of these autolytic cleavage sites in the structure of ADAMTS7, we modeled the structure of the N-terminal domains of ADAMTS7 (MP-Spacer) using the structure of ADAMTS13 as a template. This revealed that the Glu-729–Val-730 and Glu-732–Ala-733 bonds are both present in what is predicted to be the surface-exposed β3-β4 loop of the Spacer domain, which is in line with their susceptibility to proteolysis (Fig. 3). This also suggests that the autolytic reaction may have functional consequences as this Spacer region contains a substrate-binding exosite in ADAMTS13 (18–20).

### ADAMTS7 cleaves LTBP4

In addition to the autolytic cleavage fragments, TAILS identified 45 other extracellular matrix proteins that exhibited evidence of increased proteolysis in the presence of active ADAMTS7 (Table S1). To confirm that these findings were a direct consequence of proteolysis by ADAMTS7, we selected candidate substrates for further study. Selection was based on the nature of the scissile bonds, the location of the cleavage within the protein, potential functional consequences of proteolysis, and tissue distribution.

**Figure 4.**
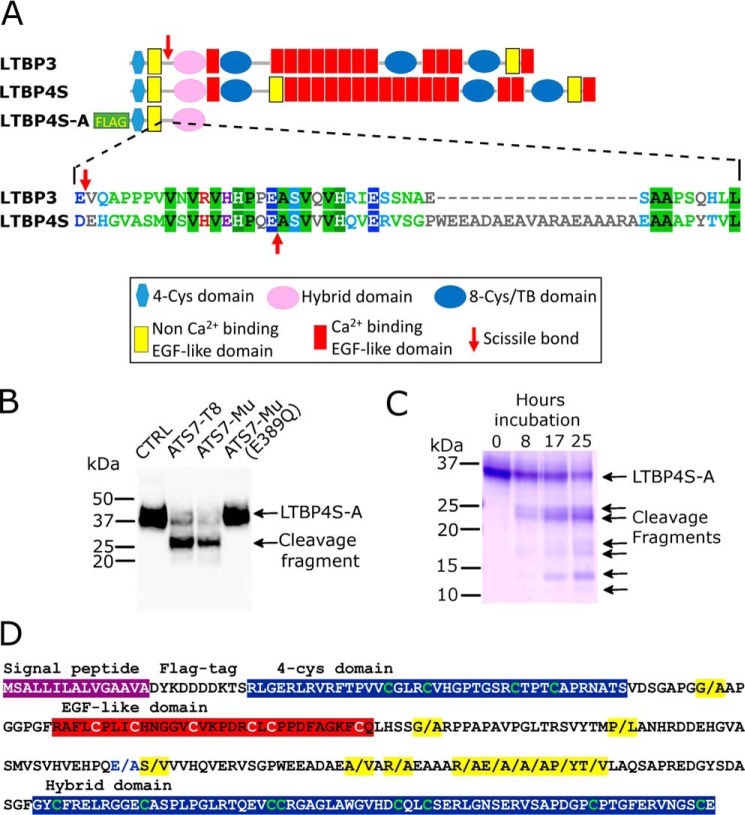
**Identification of LTBP4 as a substrate of ADAMTS7.** *A*, domain organization of LTBP3 and LTBP4S, which is the shorter isoform of LTBP4, indicating the location of the scissile bonds (*red arrow*) that were identified by TAILS. LTBP4S-A is a truncated recombinant variant that contains an N-terminal FLAG-tag. For the linker region that contains the identified scissile bonds, an alignment of the amino acid sequence of LTBP3 and LTBP4 is shown, indicating the scissile bonds (*red arrows*). *B*, Western blotting of LTBP4S-A conditioned media (anti-FLAG Ab) incubated for 17 h with buffer (*CTRL*), purified ADAMTS7-T8 (40 nm), ADAMTS7-Mu conditioned medium, or ADAMTS7-Mu(E389Q) conditioned medium. *C*, Coomassie Blue–stained SDS-PAGE gel of purified LTBP4S-A (10 μm) incubated with 10 nm purified ADAMTS7-T8 for 0, 8, 17, and 25 h. *D*, amino acid sequence of LTBP4S-A highlighting scissile bonds cleaved by ADAMTS7 (indicated by/and highlighted in *yellow*) as identified by LC-MS/MS analysis following TMT labeling of cleaved and uncleaved LTBP4S-A.

Two related proteins, the latent TGF-β–binding proteins (LTBP) 3 and 4 were cleaved at Glu-Val and Glu-Ala, respectively, matching the most prominent and confirmed autolytic cleavage sites in ADAMTS7 (Glu-729–Val-730 and Glu-732–Ala-733). A sequence alignment of the two proteins showed LTBP3 and LTBP4 were both cleaved in the same linker region, between the first EGF-like domain and the hybrid domain (Fig. 4*A*). That the cleavage appeared to occur in a linker region between domains suggested that proteolysis is expected to separate the two cleavage fragments. For LTBP4, this could potentially affect the function of LTBP4 in elastic fiber formation/organization. Physiologically, LTBP4 contributes to the deposition of tropoelastin on fibrillin-1 microfibrils (21). This occurs by binding of the N-terminal domains of LTBP4 to the fibulin-5/tropoelastin complex and binding of the C-terminal domains of LTBP4 to fibrillin-1 (22). Cleavages by ADAMTS7, therefore, have the potential to separate the fibulin-5/elastin–binding region from its fibrillin-1–binding region, thereby disrupting its essential bridging function. Importantly, LTBP4 is also co-expressed with ADAMTS7 in the adult heart and lung (23).

For these reasons, we decided to further investigate the apparent proteolysis of LTBP4 by ADAMTS7. An N-terminal fragment of LTBP4 (LTBP4S-A) that lacks domains C-terminal of the hybrid domain was expressed in HEK293T cells and the conditioned medium incubated with ADAMTS7-T8, ADAMTS7-Mu, or the inactive variant ADAMTS7-Mu(E389Q). Analysis by nonreducing Western blotting showed LTBP4S-A cleavage when incubated with ADAMTS7-Mu or ADAMTS7-T8 but not with the inactive control ADAMTS7-Mu(E389Q) (Fig. 4*B*). Coomassie Blue–stained SDS-PAGE analysis of purified LTBP4S-A cleaved by 10 nm purified ADAMTS7-T8 showed multiple cleavage fragments after 8-h incubation (Fig. 4*C*). These results show that LTBP4 is cleaved by low nanomolar concentrations of ADAMTS7 and is therefore a potential physiological substrate.

The presence of multiple bands following digestion of LTBP4 by ADAMTS7 suggests the presence of additional cleavage sites. Therefore, cleaved and uncleaved LTBP4S-A were labeled with tandem mass tags (TMT) at the N termini and analyzed by LC-MS/MS. TMT-labeled peptides (Table S3) revealed a total of 12 scissile bonds, predominantly containing hydrophobic residues Ala, Val, and Leu in P1′ (Fig. 4*D*), strongly suggesting these are the preferred residues in P1′ for ADAMTS7. P1 residues included Glu, but also Arg, Ala, Pro, and Gly.

### TIMP-4 is an efficient inhibitor of ADAMTS7

**Figure 5.**
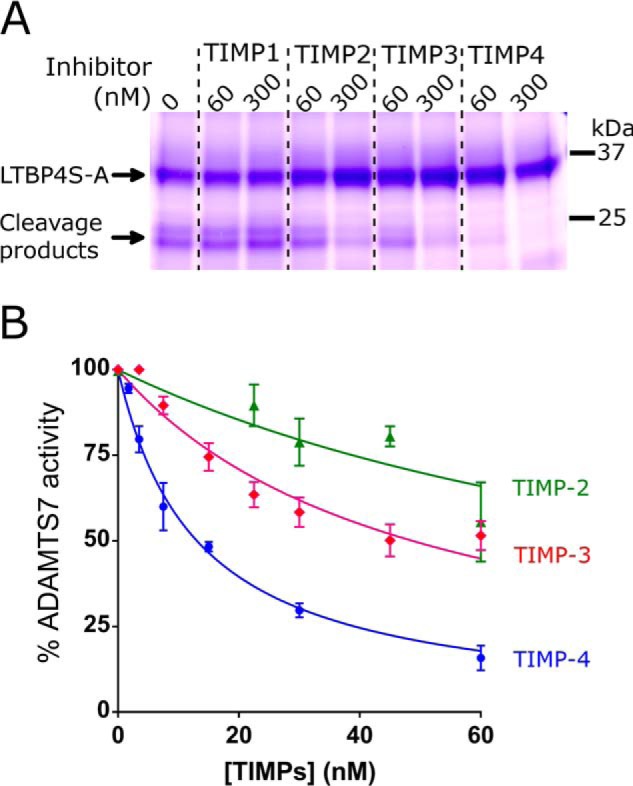
**Inhibition of ADAMTS7 by tissue inhibitors of metalloproteinases (TIMP).** *A*, ADAMTS7-T8 (9 nm) was incubated with LTBP4S-A (20 μm) at 37 °C for 17 h in the absence or presence of 60 or 300 nm TIMP1, 2, 3, or 4 and proteolysis was monitored by SDS-PAGE/Coomassie Blue. *B*, various concentrations (1.75–60 nm) of TIMP-2 (*green triangles*), TIMP-3 (*pink diamonds*) or TIMP-4 (*blue circles*) were incubated with 18 nm ADAMTS7 and 10 μm LTBP4S-A for 17 h and inhibition of proteolysis was monitored by densitometry of LTBP4S-A on SDS-PAGE/Coomassie Blue. The derived *K_i_*(_app_) for TIMP-4 was 13 nm, compared with 49 nm for TIMP-3 and >100 nm for TIMP-2. Data represent average ± S.E. (*n* = 3).

Inhibition of ADAMTS7 by endogenous metalloprotease inhibitors (TIMPs) has not been investigated. We therefore investigated if LTPB4S-A cleavage by ADAMTS7 can be specifically inhibited by TIMP members. This revealed that of the four TIMPs, TIMP-4 is the most potent inhibitor of ADAMTS7 (Fig. 5*A*). TIMP-3 showed moderate inhibition and TIMP-1 and TIMP-2 little to no inhibition. To quantify these differences, proteolysis was monitored by densitometry of LTBP4S-A on SDS-PAGE/Coomassie Blue. This showed that in this assay the apparent inhibition constant *K_i_*(_app_) for TIMP-4 was 13 nm, compared with 49 nm for TIMP-3 and >100 nm for TIMP-2 (Fig. 5*B*). These findings confirm that for ADAMTS7, contrary to other ADAMTS family members, TIMP-4 is the most potent inhibitor.

## Discussion

Recently, ADAMTS7 has emerged as a modifier of CAD (7, 24). However, its substrate specificity is poorly defined and knowledge of cleavage sites is required to understand its physiological role. Here, for the first time, we report scissile bonds targeted by ADAMTS7. These include the autolytic cleavage sites Glu-732–Ala-733 and Glu-729–Val-730. We also identified LTBP4 as a novel substrate for ADAMTS7. In LTBP4, the P1′ residues were also predominantly Ala and Val, suggesting they are favored in P1′. Several different LTBP4 residues were found in P1, including Glu, Ala, Arg, and Pro. However, mutagenesis of the ADAMTS7 residues Glu-729 and Glu-730 to Ala reduced autolysis, which shows that ADAMTS7 prefers Glu over Ala at the P1 position. Together, these results shed light on the previously unknown cleavage site specificity of ADAMTS7. This may also benefit the development of small molecule inhibitors, which generally target the so-called specificity pocket (S1′) in metalloproteases (25). It has been suggested that small molecule inhibitors of ADAMTS7 could have therapeutic potential in CAD (24, 26) given that A*damts7*^−/−^ mice have reduced atherosclerosis in hyperlipidemic mouse models (7). Importantly, the identified cleavage sites in LTBP4 allow the development of neo-epitope antibodies to study whether proteolysis occurs at these sites *in vivo*. Evidence that this may be the case comes from a proteomic analysis of human aorta which identified LTBP4 peptides derived from cleavage events at the sites we identified (E-195↓Ala-196 and Ala-196↓Ala-197) (27, 28).

We showed that ADAMTS7 cleaves LTBP4 in a way that separates the fibulin-5/elastin–binding region from its fibrillin-1–binding region and could, therefore, affect the deposition of tropoelastin on fibrillin-1 microfibrils (21, 22). Whether this occurs in physiological and/or pathological situations needs further investigation. However, ADAMTS7 and LTBP4 have overlapping tissue expression patterns, most notably in cardiac and lung tissue (7, 23), suggesting this could be the case. The enzyme concentration at which we detect proteolysis (low nanomolar) also suggests that this could be physiologically relevant. Interestingly, several other ADAMTS and ADAMTS-like proteins have been implicated in fibrillin-1 microfibril biology, including ADAMTS10, ADAMTS17, and ADAMTS6 (30–33). Phylogenetic analysis of ADAMTS genes shows that ADAMTS7 and 12 are more closely related to ADAMTS17, 19, 6, and 10 than to the other 13 ADAMTS family members (34), which may indicate a functional relationship, like that between ADAMTS1, 4, and 5, which are all are all involved in versican and aggrecan turnover.

Autolysis has been reported for several ADAMTS family members (3, 35–37). For ADAMTS4 and ADAMTS5, this also involved cleavages in the Spacer domain (35, 36). Here, we show that cleavage of the Glu-732–Ala-733 and Glu-729–Val-730 in the ADAMTS7 Spacer domain depends on ADAMTS7 activity. Our LTBP4 cleavage data further confirmed that ADAMTS7 is capable of cleaving Glu-Ala bonds. These findings strongly suggest that ADAMTS7, like other ADAMTS family members, can cleave itself. Whether this occurs *in vivo* needs further investigation. Autolysis of ADAMTS proteins has so far only been demonstrated in conditioned media and purified ADAMTS proteases. Where conditioned media was used to demonstrate autolysis, the involvement of proteolytic cascades cannot be completely ruled out.

It has previously been shown that ADAMTS activity can be regulated by TIMPs. This has so far only been shown for TIMP-3, which inhibits ADAMTS4 and 5 efficiently (14, 38). TIMP-3 inhibits ADAMTS2 very poorly (14) and ADAMTS13 and ADAMTS15 are not inhibited by any of the TIMPs (39). TIMP-4 does not inhibit ADAMTS1, 4, or 5 efficiently (38, 40, 41). Surprisingly, we showed that TIMP-4 is the most efficient inhibitor of ADAMTS7, although TIMP-3 also showed inhibitory activity. Importantly, TIMP4 inhibited ADAMTS7 efficiently at low nanomolar concentrations, suggesting that this could potentially function as the endogenous inhibitor of ADAMTS7. Both TIMP-4 and ADAMTS7 have a restricted tissue distribution with particularly abundant expression in adult cardiac tissue (42, 43), which is therefore a likely site of physiological activity and regulation. Interestingly, *Timp4*^−/−^ mice are more vulnerable to myocardial infarction (44). Our findings suggest unregulated ADAMTS7 may potentially contribute to this phenotype.

Our TAILS analysis identified 45 candidate substrates of ADAMTS7, and in this study we confirmed proteolysis of LTBP4 and autolysis. Whether the other ECM proteins identified are susceptible to proteolysis by ADAMTS7 needs confirmation. One of these, LTBP3, appeared to be cleaved in the same N-terminal linker region as LTBP4. However, the physiological consequence of LTBP3 proteolysis is not immediately clear. The primary function of LTBP3 is reportedly to influence the bioavailability of TGFβ, in cartilage and bone in particular (45). The high-MW, latent forms of TGFβ1–3 bind to the middle 8-Cys/TB domain of LTBP3. Mutations in LTBP3 cause dental anomalies and short stature but also appear to increase risk of thoracic aortic aneurysms and dissections (46, 47). Binding sites on LTBP3 for fibrillin-1 and/or other ECM molecules have not been identified, suggesting that the effect of proteolysis may be distinct from the effect on LTBP4.

Interestingly, LTBP1 was also identified as a potential ADAMTS7 substrate. LTBP1 exists in two major forms: long (L) and short (S), which are transcribed from independent promoters and differ in their N terminus only (48). The apparent cleavage site, as suggested by our TAILS analysis, is present in the long form (Lys-318–Gly-319), but not in the short form. Although the potential consequence of proteolysis at this site needs characterization, it is interesting that the long form of LTBP1 (LTBP1L) is primarily important for cardiac and cardiac valve development (49, 50).

For COMP, previously reported to be susceptible to proteolysis by ADAMTS7 (15), our TAILS analysis did not identify cleavage products. COMP was expressed by the cells, as the peptide containing the N terminus of secreted COMP (following removal of the signal peptide) was identified. Using recombinant COMP, we previously found that the ADAMTS7 concentration required for proteolysis of COMP is >500 nm (51), which likely exceeds the concentration of ADAMTS7 in our TAILS experiment and is much higher than what is required to cleave LTBP4 (<10 nm).

In conclusion, we identified and confirmed a novel substrate of ADAMTS7, LTBP4, the cleavage of which has the potential to impact on elastogenesis, and consequently, elasticity in the cardiovascular system. We shed light on the previously unknown cleavage site specificity of ADAMTS7 and identified TIMP-4 as an efficient inhibitor, which makes it a likely cardiovascular regulator of ADAMTS7. The autolytic reactions that we report here, may provide an additional mode of regulation. Several other candidate substrates that were identified by TAILS, are of potential interest and require further investigation.

## Experimental procedures

### Generation of expression vectors

Human ADAMTS7 cDNA was obtained from Source Bioscience (I.M.A.G.E. clone 6650221). The cDNA, including the native signal peptide, was cloned into the pcDNA3.1myc/His expression vector, which adds an myc and His_6_ tag at the C terminus. Truncated ADAMTS7 variants were truncated after the following amino acids: Pro-997 (T4), Gly-1414 (Mu), and Pro-1630 (T8). Point mutants were generated by site-directed mutagenesis (KOD Hotstart, Merck). The ADAMTS7-T4 FCSM contains the mutations R68A/R70A/R232A/R236A. The coding sequence of all constructs that were generated was verified by Sanger sequencing (Genewiz UK Ltd). The mammalian expression vector for LTBP4S-A was a generous gift of Tomoyuki Nakamura (Kansai Medical University, Osaka, Japan) and details of its generation have been described previously (21).

### iTRAQ-TAILS

The conditioned medium (serum-free Dulbecco's modified medium) of human skin fibroblasts co-cultured with HEK cells stably expressing ADAMTS7-Mu or inactive ADAMTS7-Mu(E389Q) was prepared for proteomic analysis by LC-MS/MS as previously described (52). Briefly, for each experimental condition, 3.5 × 10^7^ HEK cells and 3.3 × 10^7^ fibroblasts were co-cultured in serum-free Dulbecco's modified medium supplemented with amino acids and vitamin C. 120 ml of conditioned medium was harvested per condition and protease inhibitors AEBSF (0.1 mm) and EDTA (1 mm) were added. The conditioned medium was concentrated 100× and 500 μg of total protein was denatured with 2.5 m GuHCl, prior to reduction with 1 mm tris(2-carboxyethyl)phosphine) at 65 °C for 45 min. Cysteines were alkylated with 5 mm iodoacetamide for 1 h at room temperature in the dark. Free amines, including those of neo–N termini generated by ADAMTS7, were labeled with iTRAQ® labels. Proteins recovered from the “active protease condition” and from the “inactive control condition” were labeled with 2.5 mg iTRAQ® 115 or iTRAQ® 117, respectively. After trypsinization with Trypsin Gold (Promega), most peptides not labeled with iTRAQ were removed by coupling to the amine reactive polymer HPG-ALD (Flintbox) and centrifugal filtration (Amicon Ultra 10 kDa MWCO). Mass spectrometry was performed with an ESI-Q Exactive mass spectrometer (coupled to 2D-RP/RP NanoAcquity UPLC) at the Proteomic Facility of the University of Liège. For data analysis, the open source Trans-Proteomic Pipeline (TPP) was used. Peptides of potential interest (Table S1) were selected based on labeling at the N terminus with iTRAQ and overrepresentation in the protease condition (>1.5×) *versus* control condition. Excluded were peptides that mapped to native N termini or N termini immediately following signal peptides or furin cleavage sites. Also excluded were peptides that mapped to proteins that are not secreted.

### Expression and purification of ADAMTS7 and LTBP4

ADAMTS7, ADAMTS7 variants, and LTBP4S-A were transiently expressed in HEK293T cells in Opti-MEM (Invitrogen) using PEI MAX 40K (Polysciences, Inc.) as a transfection reagent. HEK cells stably expressing ADAMTS7-Mu, ADAMTS7-Mu(E389Q), and ADAMTS7-T8 were generated using G418 (Sigma-Aldrich) as a selection reagent. ADAMTS7-T8 was purified using anion exchange chromatography and gel filtration (53). Briefly, 1 liter conditioned medium was loaded at pH 7.8, followed by washing of the column with 705 mm NaCl, 20 mm Tris, pH 7.8, 10 mm CaCl_2_ and eluting with 2 m NaCl, 20 mm Tris, pH 7.8, 10 mm CaCl_2_. Gel filtration chromatography was employed using a HiPrep Sephacryl S-200 HR column for further purification and exchanging the buffer into 20 mm Tris, 150 mm NaCl, 10 mm CaCl_2._ Protein purity was assessed by SDS-PAGE/Coomassie Blue staining. Eluted fractions were concentrated 5× using Amicon Ultra centrifugal filter units (MWCO 100 kDa) and the purified enzyme was aliquoted and stored at −80 °C until use in proteolytic activity assays. LTBP4S-A was purified using ANTI-FLAG® M2 Affinity Gel (Sigma-Aldrich) using FLAG peptide (Sigma-Aldrich) for elution.

### SDS-PAGE and Western blotting

For SDS-PAGE analysis, Bolt^TM^ 4–12% (ADAMTS7) or 12% (LTBP4S-A) Bis-Tris Plus Gels (Thermo Fisher) were used. Samples were reduced with 5% β-mercaptoethanol where indicated. ADAMTS7 and ADAMTS7 variants were detected with anti-myc Ab (9E10, Santa Cruz Biotechnology). LTBP4S-A was recognized with anti-FLAG (OctA probe) Ab sc-66355 (Santa Cruz Biotechnology). All primary antibodies were used at 0.2 μg/ml in phosphate-buffered saline (PBS), 5% nonfat dried milk powder and detected with appropriate horseradish peroxidase (HRP)–labeled secondary Ab (DAKO). Immobilon Chemiluminescent HRP substrate (Merck Millipore) was detected with a ChemiDoc Touch Imaging System (Bio-Rad). For Coomassie Blue staining, gels were stained for 2 h with Thermo Fisher Imperial protein stain and destained in water overnight at room temperature on an orbital shaker. Treatment of ADAMTS7-T4 CM with recombinant human furin (PeproTech) was performed at a final furin concentration of 50 nm.

### N-terminal sequencing of ADAMTS7 autolytic product

ADAMTS7-T4 was expressed at a large scale (0.5 liter), purified using a Ni^2+^-chelating column (HiTrap, GE Healthcare), and eluted using an imidazole gradient (20–250 mm). Fractions containing ADAMTS7-T4 and its C-terminal autolytic product were identified using dot blot with the anti-Myc Ab (Santa Cruz Biotechnology). Protein purity of these samples was assessed by SDS-PAGE/silver staining. Samples were dialyzed against 20 mm Tris, pH 7.5, 150 mm NaCl, 5 mm CaCl_2,_ and concentrated 10× using Amicon Ultra centrifugal filter units, MWCO 10 kDa (Merck). ADAMTS7-T4 and its C-terminal autolytic product were separated by SDS-PAGE using Bolt^TM^ 4–12% Bis-Tris Plus Gels (Thermo Fisher) and transferred to PVDF (Millipore Immobilon PSQ) with Bolt Transfer Buffer (Thermo Fisher) and Trans-Blot® Turbo^TM^ Transfer Instrument (Bio-Rad). Protein bands were visualized with Ponceau S staining (Amresco) and the 45-kDa autolytic product band was excised and sent to Alphalyse A/S (Denmark) for N-terminal sequencing.

### Molecular modeling of ADAMTS7

The structure of the N-terminal domains (MP-Spacer) of ADAMTS7 was modeled with the Bioinformatics Toolkit of the Max Planck Institute for Developmental Biology, Tübingen, Germany (54, 55). The templates used for modeling were the ADAMTS structures 3GHM, 2RJQ, 2RJP, 4WK7, and 2V4B. Molecular graphics were produced with an open source version of PyMOL precompiled by Christoph Gohlke (University of California, Irvine).

### LTBP4S-A cleavage assays

Purified LTBP4S-A (10 μm) was incubated at 37 °C with or without the indicated concentration of purified ADAMTS7-T8 in 20 mm Tris, pH 7.5, 150 mm NaCl, 10 mm CaCl_2_ for the specified period of time. The indicated concentrations of purified ADAMTS7-T8 used in the assays are the concentration of active protease as determined by active-site titration with TIMP-4. Where TIMPs were used in the assay, recombinant human TIMP1, 2, 3, or 4 (R&D Systems) were pre-incubated with ADAMTS7-T8 for 1 h at 37 °C. Proteolysis was stopped by addition of Bolt^TM^ LDS Sample Buffer, 5% β-mercaptoethanol, and heating to 95 °C. Samples were frozen at −20 °C until analysis by SDS-PAGE/Coomassie Blue. To measure TIMP-4 inhibition, proteolysis was quantified by densitometry using ImageJ.

### Active site titration of ADAMTS7-T8

TIMP-4 was titrated into the LTBP4SA cleavage assays at concentrations ranging from 1.75 nm to 60 nm, and relative activity was plotted against TIMP-4 concentration to establish the concentration of active protease in purified ADAMTS7-T8 stock solutions retrospectively (29).

### Identification of LTBP4S-A cleavage sites

Purified LTBP4S-A (80 μg) was incubated for 25 h with 70 nm purified ADAMTS7-T8 in 50 mm HEPES, pH 7.5, 5 mm CaCl_2_ in the presence or absence of broad-spectrum metalloprotease inhibitor GM6001 (90 μm). Samples were analyzed by SDS-PAGE/Coomassie Blue to confirm proteolysis and the absence thereof in the cleavage and control condition, respectively. New N termini generated by ADAMTS7 were labeled with Tandem Mass Tags (Thermo Fisher) according to the manufacturer's instructions prior to incomplete digest with trypsin (Thermo Fisher), chymotrypsin (Thermo Fisher) and Glu-C (Thermo Fisher). LC-MS/MS was performed at the Proteomic Facility of the University of Liège using an ACQUITY UPLC M-Class System (Waters) hyphenated to a Q Exactive (Thermo Scientific), in nanoelectrospray positive ion mode. Data were analyzed with Proteome Discoverer version. 2.1.1.21. The protein/peptide identifications were performed against a bovine background protein database supplemented with the sequence of the human target protein LTBP4. Search parameters were set as “no Enzyme” because of the specific multi-enzymatic limited digestion that was applied. Scissile bonds were derived from peptides labeled with TMT at the N terminus that were identified in the active protease condition, but not in the control condition. All reported peptides have a false discovery rate equal or lower than 0.01.

## Author contributions

A. C. and R. d. G. funding acquisition; A. C. designed experiments, analyzed data, and wrote the paper; C. M. performed experiments, analyzed data, and wrote the paper; J. T. B. C. and S. S. designed experiments, analyzed data, and wrote the paper; R. d. G. designed and performed experiments, analyzed data, prepared the figures, and wrote the paper.

## Supplementary Material

Supporting Information

## Footnotes

2 The abbreviations used are:

MP: metalloprotease
ECM: extracellular matrix
CAD: coronary artery disease
TAILS: terminal amine isotopic labeling of substrates
MW: molecular weight
Ab: antibody
LTBP: latent TGF-β–binding proteins
TMT: tandem mass tags
FCSM: furin cleavage site mutant
COMP: cartilage oligomeric matrix protein.
