# Supplementary material for: Proteomic discovery of substrates of the cardiovascular protease ADAMTS7
Source: J Biol Chem. 2019 Mar 29;294(20):8037–45. doi: 10.1074/jbc.RA119.007492 (PMC6527163; doi:10.1074/jbc.RA119.007492)
Supplement: Supporting Information [file supp_294_20_8037__index.html]

Proteomic discovery of substrates of the cardiovascular protease ADAMTS7 — ADAMTS7 substrate specificity — Proteomic discovery of substrates of the cardiovascular protease ADAMTS7 — ADAMTS7 substrate specificity — Supporting Information 

# Proteomic discovery of substrates of the cardiovascular protease ADAMTS7

## Supporting Information

- Supporting Information (to be published online) - Tables S1, S2, S3
