# Supplementary material for: Proteomic discovery of substrates of the cardiovascular protease ADAMTS7
Source: J Biol Chem. 2019 Mar 29;294(20):8037–45. doi: 10.1074/jbc.RA119.007492 (PMC6527163; doi:10.1074/jbc.RA119.007492)
Supplement: Supporting Information [file supp_RA119.007492_143046_2_supp_307582_pp2sg8.pdf]

Supporting Information for:

Proteomic substrate discovery of the cardiovascular protease ADAMTS7

A. Colige[a], C. Monseur[a], J.T.B. Crawley[b], S. Santamaria[b], R. de Groot[b]

[a] Laboratory of Connective Tissue Biology
GIGA, University of Liège
Sart-Tilman
4000 Liège
Belgium

[b] Centre for Haematology
Imperial College London
W12 0NN London
United Kingdom

This PDF file includes:

Table S1: Candidate substrates of ADAMTS7 identified by TAILS

Table S2: ADAMTS7 peptides identified by TAILS suggesting autolysis

Table S3: LTBP4 scissile bonds cleaved by ADAMTS7

Corresponding Author

Dr Rens de Groot
Imperial College London
Centre for Haematology
5th Floor Commonwealth Building
Hammersmith Hospital Campus
Du Cane Road, W12 0NN London
United Kingdom
T: ++44 (0) 20 83 83 22 98
E: R.deGroot@imperial.ac.uk

Table S1. Candidate substrates of ADAMTS7 identified by TAILS

Secreted and transmembrane proteins containing neo-N-termini that were enriched in the presence of active recombinant ADAMTS7 vs inactive ADAMTS7(E389Q). Listed is 1) the ID of the protein in the UniProt database, 2) the full name of the protein, 3) whether the protein is a secreted (S) or a transmembrane (TM) protein, 4) the P1 residue preceding the identified peptide, 5) the amino acid sequence of the identified peptide, 6) the intensity ratio of iTRAQ labels originating from the protease (P) and control (C) condition and 7) the location of the new N-terminus in the structure of the identified protein. ADAMTS7 peptides are listed separately in Table S2.

UniProt ID	Protein name	TM/S	P1	Peptide	P:C	Location new N-terminus
O95450	A disintegrin and metalloproteinase with thrombospondin motifs 2	S	A42	ADPPGGPLGHGAER	1.9	Prodomain
O00468	Agrin	S	K1863	SAGDVDTLAFDGR	1.6	Loop connecting EGF-like 4 and Laminin G-like 3 domains
P22004	Bone morphogenetic protein 6	S	A35	AAAAGGQLLGDGGSPGR	2.4	Propeptide
Q9NYQ6	Cadherin EGF LAG seven-pass G-type receptor 1	TM	R62	ELLDVGR	1.6	Unknown structure
O75976	Carboxypeptidase D	S	G227	EPPALDEVPEVR	3.4	Loop in Peptidase M14 domain
P16870	Carboxypeptidase E	S	R42	LQQEDGISFEYHR	1.7	N-terminus preceding Peptidase M14 domain
P07858	Cathepsin B	S	K79	LPASFDAR	1.7	Loop connecting propeptide with cysteine peptidase domain
P10909	Clusterin	S	R325	ELDESLQVAER	1.8	Clusterin domain
P10909	Clusterin	S	A266	FQHPPTEFIR	1.8	Clusterin domain
P10909	Clusterin	S	P265	AFQHPPTEFIR	1.5	Clusterin domain
P02452	Collagen alpha-1(I) chain	S	A1218	DDANVVR	1.6	Loop preceding C-terminal prodomain. (BMP1 cleavage site)
P02452	Collagen alpha-1(I) chain	S	G174	ISVPGPMGPSGPR	1.5	Non-helical region following the n-terminal propeptide
P02452	Collagen alpha-1(I) chain	S	K781	GESGPSGPAGPTGAR	1.6	Triple-helical region
P02452	Collagen alpha-1(I) chain	S	R1217	ADDANVVR	1.5	Loop preceding C-terminal prodomain
P02461	Collagen alpha-1(III) chain	S	N1251	GQIESLISPDGSR	1.6	C-terminal propeptide (Stalk region)
P20908	Collagen alpha-1(V) chain	S	P435	ANQDTIYEGIGGPR	1.9	Nonhelical region
P39060	Collagen alpha-1(XVIII) chain	S	A1533	DDILASPPR	2.1	NC1 hinge region. Releases endostatin.
P08123	Collagen alpha-2(I) chain	S	P983	SGPVGPAGAVGPR	1.6	Triple helical region
P08572	Collagen alpha-2(IV) chain	S	G1353	FMGNTGPTGAVGDR	1.9	Triple-helical region
P05997	Collagen alpha-2(V) chain	S	G213	SVGPVGPR	2.7	Triple-helical region
P12111	Collagen alpha-3(VI) chain	S	G2240	LIGEQGISGPR	2.2	Collagen-like region
P01034	Cystatin-C	S	R34	LVGGPMDASVEEEGVR	3.2	N-terminal region. Proteolysis may affect inhibitory function.
Q9NT22	EMILIN-3	S	V224	GFGVIPEGLVGPGDR	2.0	Unknown structure
P54756	Ephrin type-A receptor 5	TM	A55	SPSNEVNLLDSR	1.7	Prior to Eph LBD domain
Q8IWU6	Extracellular sulfatase Sulf-1	S	N558	LEEEEELQVLQPR	4.8	PFAM predicted domain DUF3740
Q8IWU6	Extracellular sulfatase Sulf-1	S	Y555	DINLEEEEELQVLQPR	2.4	PFAM predicted domain DUF3740
Q8IWU5	Extracellular sulfatase Sulf-2	S	Y549	HVGLGDAAQPR	2.2	PFAM predicted domain DUF3740
P35556	Fibrillin-2	S	N781	GICENLR	1.6	EGF-like 11 domain
P35556	Fibrillin-2	S	R48	SATAGSEGGFLAPEYR	2.5	Propeptide
P02751	Fibronectin	S	T279	SSGSGPFTDVR	1.7	Loop connecting Fibronectin type-I 5 and Fibronectin type-I 6 domains
P02751	Fibronectin	S	S281	GSGPFTDVR	4.8	Loop connecting Fibronectin type-I 5 and Fibronectin type-I 6 domains
Q86VR8	Four-jointed box protein 1	S	L84	TLAAGADGPPR	1.5	Unknown structure
P22466	Galanin peptides	S	L109	DLPAAASSEDIER	2.7	Galanin message associated peptide (GMAP)
Q08380	Galectin-3-binding protein	S	K441	YSSDYFQAPSDYR	2.2	Unknown structure

Q08380	Galectin-3-binding protein	S	R128	STHTLDLSR	1.8	Loop connecting SRCR and BTB domains
Q08380	Galectin-3-binding protein	S	Y446	FQAPSDYR	2.7	Unknown structure
P28799	Granulins	S	A201	LSSSVMCPDAR	1.5	Loop connecting granulin F and Granulin B
P28799	Granulins	S	S203	SSVMCPDAR	1.5	Loop connecting granulin F and Granulin B
P28799	Granulins	S	S204	SVMCPDAR	1.7	Loop connecting granulin F and Granulin B
P28799	Granulins	S	S205	VMCPDAR	1.7	Loop connecting granulin F and Granulin B
P30443	HLA class I histocompatibility antigen, A-1 alpha chain	TM	V49	GYVDDTQFVR	1.8	Alpha-1 region
P08476	Inhibin beta A chain	S	N98	GYVEIEDDIGR	1.7	Propeptide
P05019	Insulin-like growth factor I	S	A48	GPETLCGAELVDALQFVCGDR	>>1	Loop connecting N-terminal propeptide with IGF I
Q08431	Lactadherin	S	N34	GGLCEEISQEVR	1.6	EGF-like domain
P25391	Laminin subunit alpha-1	S	C873	LGNTDGAHCER	8.1	Laminin EGF-like 8
Q14766	Latent-transforming growth factor beta-binding protein 1	S	K318	GISGEQSTEGSFPLR	2.6	Prior to 4-Cys domain. Present in long form only.
Q9NS15	**Latent-transforming growth factor beta-binding protein 3**	S	E220	VQAPPPVVNVR	1.9	Loop connecting EGF-like domain and hybrid domain
Q8N2S1	**Latent-transforming growth factor beta-binding protein 4**	S	E229	ASVVVHQVER	2.1	Loop connecting EGF-like domain and hybrid domain
Q68D85	Natural cytotoxicity triggering receptor 3 ligand 1	TM	P110	GIQLEEAGEYR	1.8	Ig-like V-type domain
O95502	Neuronal pentraxin receptor	TM	C21	IIASVPLAASPAR	2.2	Loop connecting Signal-anchor with Extracellular region
P14543	Nidogen-1	S	N233	GAYNIFANDR	1.6	NIDO (G1) domain
P07602	Prosaposin	S	N194	GDVCQDCIQMVTDIQTAVR	1.9	Loop connecting propeptide with Saposin B-type 2 domain
O15354	Prosaposin receptor GPR37	TM	R73	EEQGAAFLAGPSWDLPAAPGR	1.8	Extracellular region 1
O75629	Protein CREG1	S	R45	LPPLPPR	1.9	Loop preceding β-barrel
Q92520	Protein FAM3C	S	R41	SALDTAAR	1.6	Known maturation site removing N-terminus
Q04900	Sialomucin core protein 24	TM	T54	TPAPETCEGR	1.7	Extracellular region
O00391	Sulfhydryl oxidase 1	S	R645	DTGAALLAESR	1.5	Unknown structure
Q6ZMP0	Thrombospondin type-1 domain-containing protein 4 (ADAMTSL6)	S	R98	AFADHVVSAVR	2.1	200 amino acid long insert in TSP type-1 1 domain.

Table S2. ADAMTS7 peptides identified by TAILS suggesting autolysis

Neo-N-termini in ADAMTS7 itself that were enriched in the presence of active ADAMTS7 vs inactive ADAMTS7(E389Q) suggest autolysis. Listed are 1) the domain in which the new N-terminus is located 2) the P1 residue preceding the identified peptide sequence 3) the sequence of the identified peptide 4) the intensity ratio of iTRAQ labels originating from the active protease (P) and control (C) condition.

Domain	P1	Peptide	P:C
prodomain	R40	AALDIVHPVR	4.8
prodomain	R76	DAPAFYELQ	>>1
prodomain	R76	DAPAFYEL	4.8
prodomain	R76	DAPAFYELQYR	3.4
prodomain	P79	AFYELQYR	2.6
prodomain	T96	ANQHLLAPGFVSETR	3.2
prodomain	N98	QHLLAPGFVSETR	4.4
prodomain	Q99	HLLAPGFVSETR	7.8
prodomain	L101	LAPGFVSETR	4.0
prodomain	Q194	RGDSSAPSTCGVQVYPELESR	2.5
prodomain	R195	GDSSAPSTCGVQVYPELESR	6.2
prodomain	P201	STCGVQVYPELESR	22.6
prodomain	C204	GVQVYPELESR	4.8
prodomain	C204	GVQVYPELESRR	3.0
prodomain	G205	VQVYPELESR	3.7
prodomain	Q207	VYPELESR	5.8
MP	G328	GDAHPLHHDTAILLTR	2.2
MP	R370	SCSINEDTGLPLAF	3.6
Dis	H504	SKLDAAVDGTR	5.4
Dis	K506	LDAAVDGTR	29.4
cysteine-rich	N637	EYFAEKLR	4.3
cysteine-rich	D646	AVVDGTPCYQVR	3.1
cysteine rich	A647	VVDGTPCYQVR	55.2
cysteine-rich	D650	GTPCYQVR	3.6
cysteine-rich	G673	CDFEIDSGAMEDR	6.0
cysteine-rich	G673	CDFEIDSGAMEDR	3.0

Domain	P1	Peptide	P:C
spacer	E706	AEGLGYVDVGLIPAGAR	3.3
spacer	E708	GLGYVDVGLIPAGAR	4.3
spacer	G709	LGYVDVGLIPAGAR	10.3
spacer	G711	YVDVGLIPAGAR	4.8
spacer	Y712	VDVGLIPAGAR	30.0
spacer	V713	DVGLIPAGAR	3.4
spacer	**E729**	**VAEAANFLALR**	**92.2**
spacer	V730	AEAANFLALR	2.7
spacer	A731	EAANFLALR	3.5
spacer	**E732**	**AANFLALR**	**39.8**
spacer	N758	GDYQVAGTTFTYAR	5.7
spacer	E797	SNPGVHYEYTIHR	2.2
TSP type-1 4	C977	DEAQQPASEVTCSLPLCR	>>1
TSP type-1 4	P983	ASEVTCSLPLCR	18.2
mucin-like	N1194	DFPVGKDSQSQLPPPWR	4.3
mucin-like	K1357	GQPESLSPEVPLSSR	3.7
mucin-like	L1398	AEAGPPADPLVVR	2.2
mucin-like	A1399	EAGPPADPLVVR	4.2

Table S3. LTBP4 scissile bonds cleaved by ADAMTS7. Following LTBP4S-A incubation with ADAMTS7-T8 in the presence (control) or absence of a zinc-chelating small molecule inhibitor, samples were labelled with amine-reactive mass tags (TMT), which labels new N-termini generated by ADAMTS7. After subsequent incomplete digest with Trypsin, Chymotrypsin, and Glu-C, samples were analyzed by LC-MS/MS. Listed are the peptides labelled with TMT at the N-terminus that were found to be abundant in the active protease condition (no inhibitor) but completely absent in the control condition (with inhibitor). The P1 residue preceding the identified peptide is listed with the numbering of LTBP4S (UniProt ID: Q8N2S1-2). Note that using this numbering the P1 residue identified in the original TAILS experiment (Table S1) is E162.

P1	Peptide
**G73**	AAPGGPGF
**G119**	ARPPAPAVPGLTR
**P138**	LANHRDDEHGVAS
**P138**	LANHRDDEHGVASMVSVHVEHPQEASVVVHQVER
**S164**	VVVHQVER
**A184**	VARAEAAARAE
**R187**	AEAAAR
**R187**	AEAAARAEAAAP
**R193**	AEAAAPYT
**R193**	AEAAAPYTV
**E195**	AAAPYTVLAQ
**E195**	AAAPYTVLAQSAPREDG
**E195**	AAAPYTVLAQSAPREDGYSD
**A196**	AAPYTV
**A196**	AAPYTVLAQSAPR
**A196**	AAPYTVLAQSAPREDG
**A197**	APYTVLAQSAPR
**P199**	YTVLAQSAPR
**T201**	VLAQSAPR
